# The Pathophysiological Changes and Clinical Effects of Tetramethylpyrazine in ICR Mice with Fluoride-Induced Hepatopathy

**DOI:** 10.3390/molecules28124849

**Published:** 2023-06-19

**Authors:** Shuai Zhang, Yilei Zheng, Hong Du, Wei Zhang, Haohuan Li, Yangping Ou, Funeng Xu, Juchun Lin, Hualin Fu, Xueqing Ni, Li-Jen Chang, Gang Shu

**Affiliations:** 1 Department of Basic Veterinary Medicine, Sichuan Agricultural University, Chengdu 611100, China; shuaizhang711@163.com (S.Z.); duhong96@hotmail.com (H.D.); zhangwei26510c@126.com (W.Z.); lihaohuan7@163.com (H.L.); ouyang.ping@live.cn (Y.O.); funengxu@sicau.edu.cn (F.X.); juchunlin@126.com (J.L.); fuhl.sicau@163.com (H.F.); 2 Center for Veterinary Sciences, Zhejiang University, Hangzhou 310030, China; yileizheng@zju.edu.cn; 3 Animal Microecology Institute, College of Veterinary Medicine, Sichuan Agricultural University, Chengdu 611130, China; xueqinni@foxmail.com; 4 Department of Small Animal Clinical Sciences, Virginia-Maryland College of Veterinary Medicine, Blacksburg, VA 24061, USA

**Keywords:** acute fluorosis, tetramethylpyrazine, liver, oxidative damage, Nrf2

## Abstract

The excessive intake of fluoride, one of the trace elements required to maintain health, leads to liver injury. Tetramethylpyrazine (TMP) is a kind of traditional Chinese medicine monomer with a good antioxidant and hepatoprotective function. The aim of this study was to investigate the effect of TMP on liver injury induced by acute fluorosis. A total of 60 1-month-old male ICR mice were selected. All mice were randomly divided into five groups: a control (K) group, a model (F) group, a low-dose (LT) group, a medium-dose (MT) group, and a high-dose (HT) group. The control and model groups were given distilled water, while 40 mg/kg (LT), 80 mg/kg (MT), or 160 mg/kg (HT) of TMP was fed by gavage for two weeks, with a maximum gavage volume for the mice of 0.2 mL/10 g/d. Except for the control group, all groups were given fluoride (35 mg/kg) by an intraperitoneal injection on the last day of the experiment. The results of this study showed that, compared with the model group, TMP alleviated the pathological changes in the liver induced by the fluoride and improved the ultrastructure of liver cells; TMP significantly decreased the levels of ALT, AST, and MDA (*p* < 0.05) and increased the levels of T-AOC, T-SOD, and GSH (*p* < 0.05). The results of mRNA detection showed that TMP significantly increased the mRNA expression levels of Nrf2, HO-1, CAT, GSH-Px, and SOD in the liver compared with the model group (*p* < 0.05). In conclusion, TMP can inhibit oxidative stress by activating the Nrf2 pathway and alleviate the liver injury induced by fluoride.

## 1. Introduction

Fluorine (F) is a nonmetal element in nature that has been recognized as a strong oxidant because of its unique chemical structure [1]. Fluoride is a salted compound of fluorine that maintains a physiological homeostasis and is an essential component for bone mineralization and enamel formation [2,3]. It has been reported that an appropriate intake of fluoride helps with skeletal formation, the prevention of dental caries, the promotion of neuron conduction, and anti-aging [4]. Supplements of fluoride in drinking water have been used extensively worldwide for disinfection and the prevention of dental diseases [5,6,7]. However, the concentration of fluoride in drinking water for the prevention of dental caries should not exceed 0.7 mg/L [8]. Fluoride is a double-edged sword; a low dose of fluoride ingestion in drinking water helps to prevent dental caries, but the ingestion of fluoride in drinking water in excess of the doses described above can cause fluorosis and threaten human health. Fluorosis leads to skeletal disorders and arthrosis, which can result in deformities. Excessive fluoride can cause damage to various organs or systems of the body, leading to dental fluorosis, skeletal fluorosis, and other pathological changes in non-skeletal tissues; it has also been reported to cause encephalopathy, leading to impaired central nervous system function [9]. With the deepening of research on the topic, many clinical and animal experiments have found that the liver is one of the important target organs of fluorosis, and excessive fluoride can cause liver tissue damage [10,11]. It is known that fluorosis is an irreversible and major public health threat in China [4]. There are three potential activities that result in exposure to fluoride, including drinking water, burning coal, and drinking tea [12]. Fluorosis has become an endemic disease in China because of the habits of drinking tea and uncontrolled coal burning [13]. However, there is no specific drug for the prevention and treatment of fluorosis, so it is necessary to study the prevention and treatment of fluorosis. Recently, studies in regard to the molecular mechanism of action of fluorosis and the exploration of a traditional Chinese medication to intervene in fluorosis have become popular [14].

It has been recognized recently that the Nrf2/ARE pathway plays a key role in mitigating oxidative stress during inflammation [15]. The liver is known as an important organ involved in the regulation and initiation of the Nrf2/ARE pathway [16,17]. It has been shown that the Nrf2 pathway is activated during fluoride-induced hepatopathy to alleviate oxidative stress [18]. Furthermore, studies have shown that the Nfr2 pathway plays a role in the nervous system and reproductive system of fluorosis-affected rats in their resistance to fluorosis [19,20]. Therefore, one of the aims of this study was to explore the mechanism of action and the role of the Nrf2 pathway during fluoride-induced hepatopathy.

Over the past few decades, several studies have demonstrated the benefits of using natural products to counter oxidative stress via the Nrf2/ARE pathway [17]. Tetramethylpyrazine (TMP) is an active ingredient extracted from Ligusticum chuanxiong Hort (*Ligusticum wallichii*) [21] that has proven to be an effective component with anti-inflammatory and antioxidant properties [22]. It has been reported that the antioxidant effect of TMP mainly results from the activation of the Nrf2/HO-1 pathway in the liver, which scavenges free radicals; inhibits NF-κB; enhances the expression of glutathione peroxidase (GSH-Px), superoxide dismutase (SOD), and other associated antioxidants; and promotes the antioxidant capacity of hepatocytes [23,24]. The use of traditional Chinese herb extracts, such as Longyanshen polysaccharides, Astragalus membranaceus extracts, stilbene glycoside, gastrodin, resveratrol, and so on, to intervene in fluorosis has been studied extensively [25]. Therefore, the second purpose of this study was to clarify the clinical effects and the mechanisms of action of TPM in treating fluoride-induced hepatopathy.

## 2. Results

### 2.1. Body Weight and Liver Index

**Figure 1:**
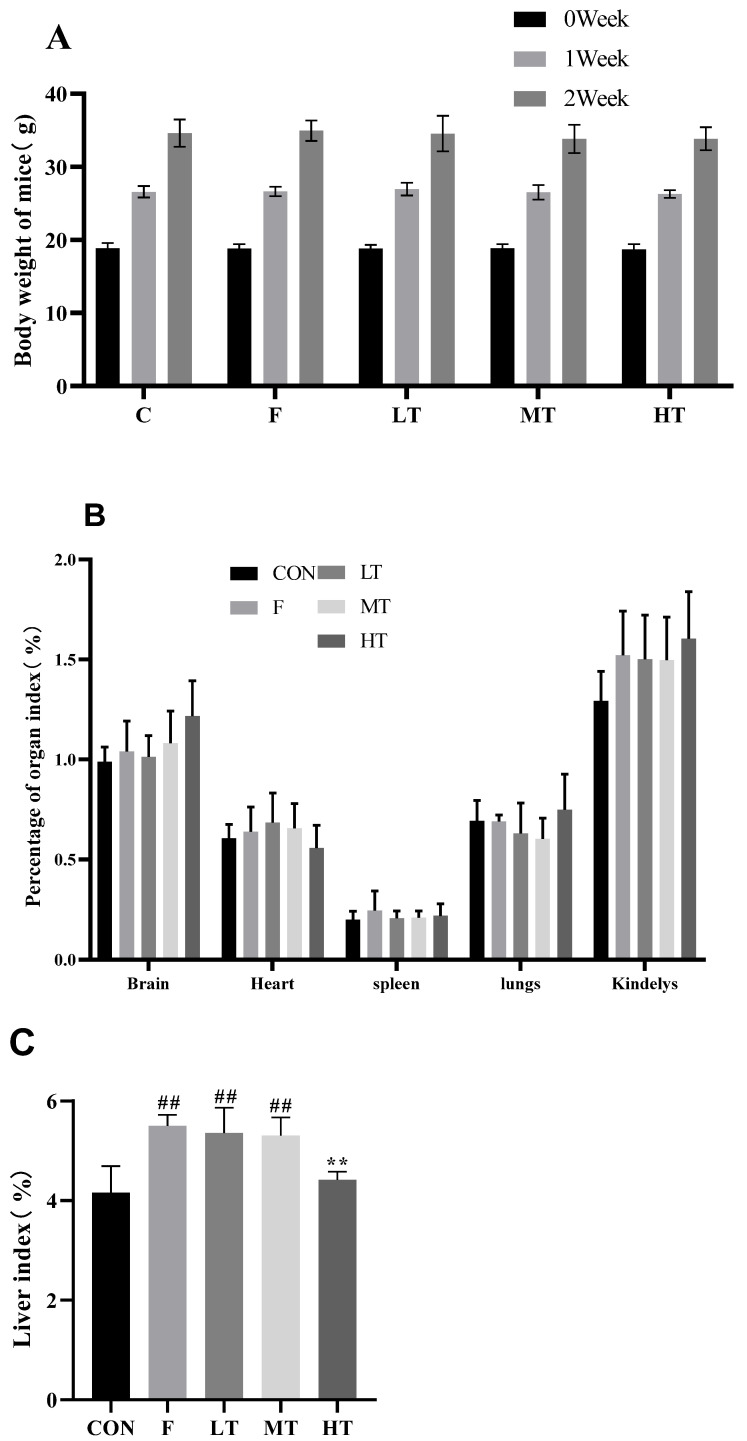
Body weights and organ indices. (**A**) Body weights of mice; (**B**) organ indices; and (**C**) organ index of liver. Data are presented as means ± SD. ** significantly different compared with group F, *p* < 0.01; *p* < 0.05; ## significantly different compared with group CON, *p* < 0.01. CON: control group; F: fluorine (alone) group; LT: low-dose TMP group; MT: medium-dose TMP group; HT: high-dose TMP group.

There were no significant differences in the body weights and organ indices except for the liver index among groups (*p* > 0.05). The organ index of the liver increased significantly in the F, LT, and MT groups (*p* < 0.05) compared to the CON group; however, the organ index of the liver in the HT group decreased significantly (*p* < 0.05) compared to group F. The results of the body weights and organ indices are shown in Figure 1.

### 2.2. Serum Levels of ALT and AST

**Figure 2:**
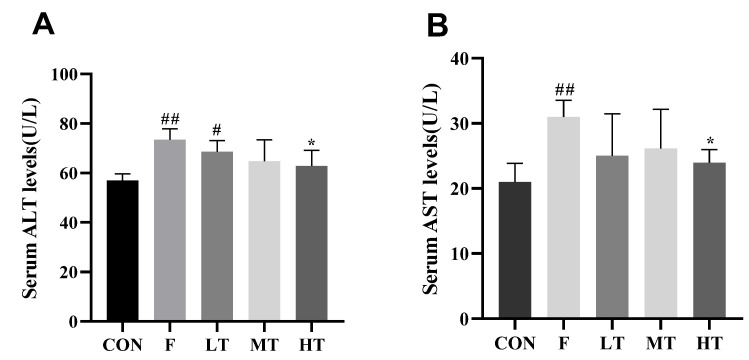
Serum levels of ALT and AST. (**A**) Serum level of ALT; (**B**) serum level of AST. * Significantly different compared with group F, *p* < 0.05; # significantly different compared with group CON, *p* < 0.05; ## significantly different compared with group CON, *p* < 0.01.

Figure 2 shows the results of the serum levels of ALT and AST. The serum levels of ALT and AST in group F were significantly higher compared to those of group CON (*p* < 0.05). The serum levels of ALT and AST showed a decreasing trend among the treatment groups compared to group F. The serum levels of ALT and AST decreased significantly in the HT group compared to group F (*p* < 0.05).

### 2.3. Serum Antioxidant Biomarkers

**Figure 3:**
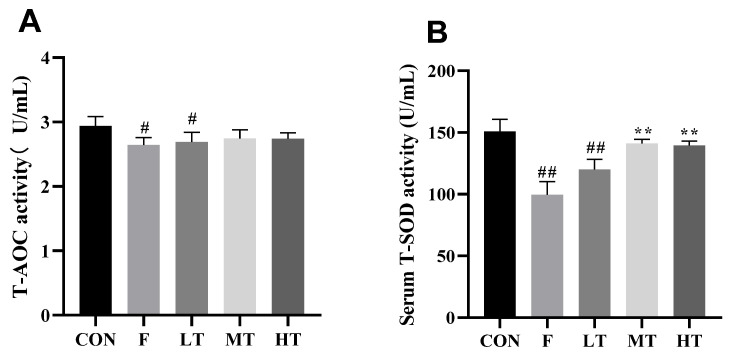

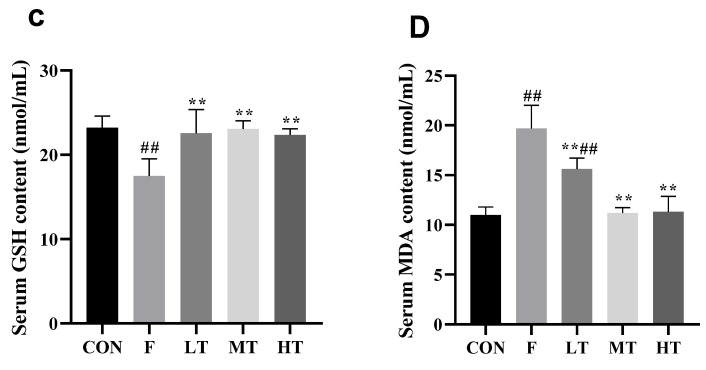
Serum antioxidant enzyme activity and oxidative concentration. (**A**) Total antioxidant capacity (T-AOC); (**B**) superoxide dismutase (T-SOD); (**C**) glutathione (GSH); and (**D**) malondialdehyde (MDA). ** significantly different compared with group F, *p* < 0.01; # significantly different compared with group CON, *p* < 0.05; ## significantly different compared with group CON, *p* < 0.01.

The results for the serum antioxidant biomarkers are shown in Figure 3. The serum levels of T-AOC, T-SOD, and GSH decreased significantly in group F compared to group CON (*p* < 0.05, *p* < 0.01); however, compared with group CON, the MDA content in group F decreased (*p* < 0.01). Nonetheless, the serum levels of T-SOD and GSH increased significantly in the HT and MT groups, whereas the content of MDA significantly decreased compared to group F (*p* < 0.01).

### 2.4. Hepatic Antioxidant Indicators

**Figure 4:**
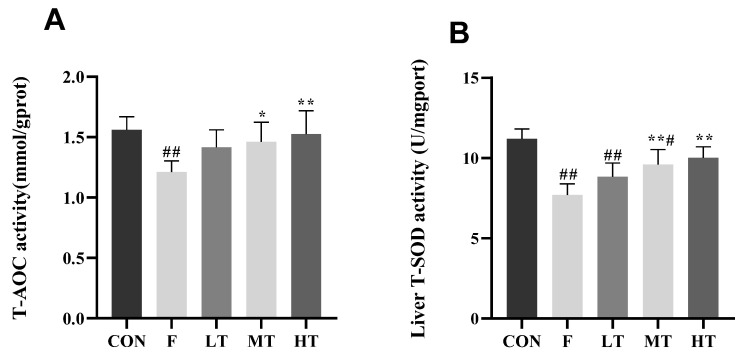

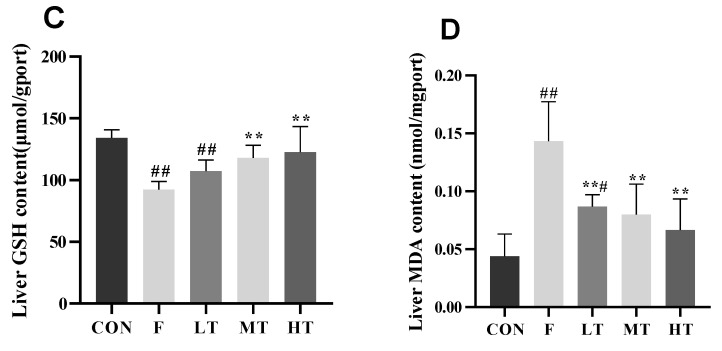
Liver antioxidant index results. (**A**) Total antioxidant capacity, T-AOC; (**B**) superoxide dismutase, T-SOD; (**C**) glutathione, GSH; and (**D**) malondialdehyde, MDA. * Significantly different compared with group F, *p* < 0.05; ** significantly different compared with group F, *p* < 0.01; # significantly different compared with group CON, *p* < 0.05; ## significantly different compared with group CON, *p* < 0.01.

The results for the hepatic antioxidant indicators are shown in Figure 4. The hepatic levels of T-AOC, T-SOD, and GSH in group F decreased significantly compared to group CON (*p* < 0.05); however, the MDA content in group F significantly increased compared to group CON (*p* < 0.01). The hepatic levels of T-AOC, T-SOD, and GSH in the MT and HT groups significantly increased, whereas the MDA content decreased compared to group F (*p* < 0.05, *p* < 0.01).

### 2.5. Hepatic Histopathological Changes

**Figure 5:**
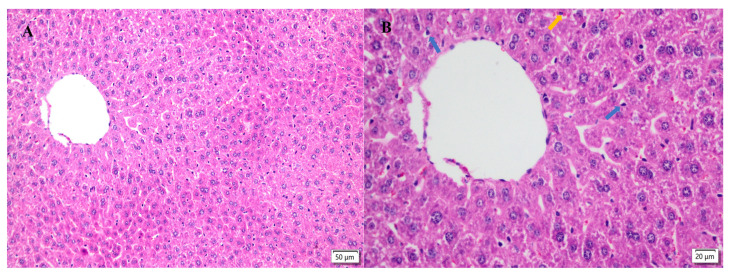

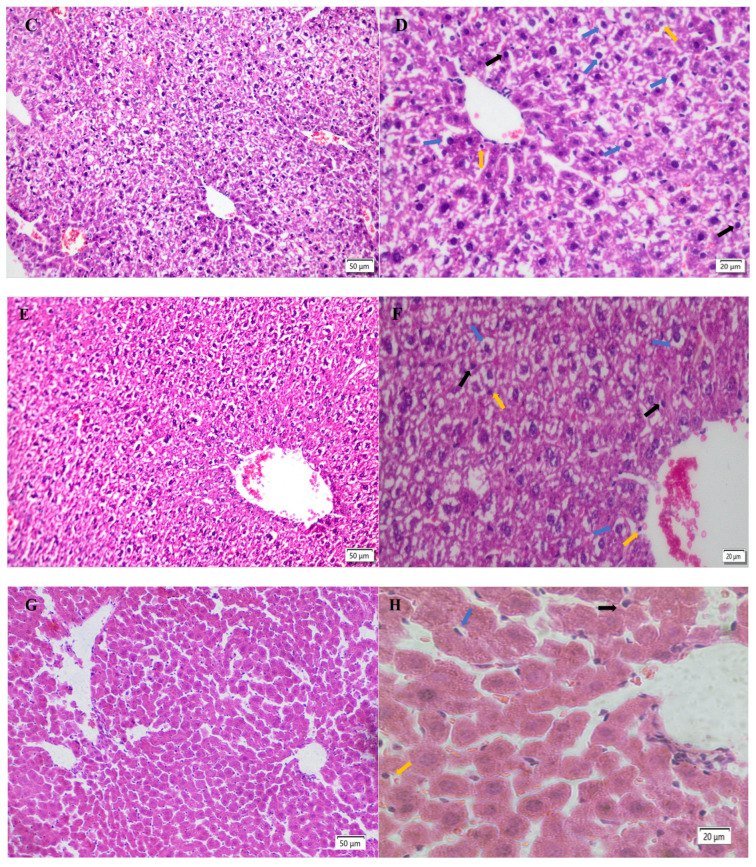

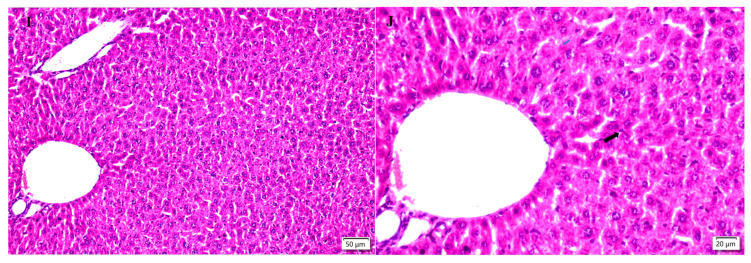
Liver morphology. Left magnification: 50 µm, 200×; right magnification: 20 µm, 400×. (**A**,**B**) The liver degeneration of the CON group. (**C**,**D**) Changes in the liver of the F group. (**E**,**F**) Changes in the liver of the LT group. (**G**,**H**) Changes in the liver of the MT group. (**I**,**J**) The liver changes of the HT group. Black arrows indicate nuclear pyknosis; blue arrows indicate vacuolar degeneration of hepatocytes; and yellow arrows indicate neutrophil infiltration.

Figure 5 presents the histopathological liver changes in this study. In the CON group, the hepatic cords were arranged tightly and orderly, the structure of central veins and hepatic sinusoids was clear, and the hepatocytes had mild vacuolar degeneration, but no obvious histopathological changes were observed (Figure 5A,B). In group F, the dilatation of central veins and hepatic sinusoids, a disordered arrangement of hepatic cords, a loose cytoplasm for hepatocytes, massive vacuolar degeneration and necrosis, and inflammatory cell infiltration were observed throughout the liver tissue (Figure 5C,D). These histopathological changes suggested successful fluorine-induced liver lesions. The pathological changes in the liver tissue were alleviated in the treatment group, and were more obvious in the MT and HT groups. The hepatic cords were closely arranged, the hepatic lobular boundaries were clear, and the structure of the hepatocytes was normal (Figure 5G–J), suggesting that TMP alleviated the fluorine-induced liver lesions.

### 2.6. Hepatic Ultrastructural Changes

**Figure 6:**
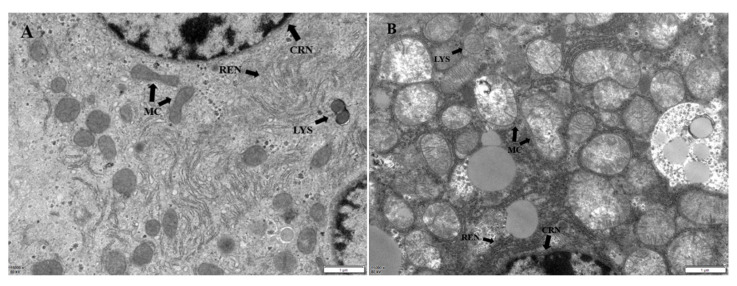

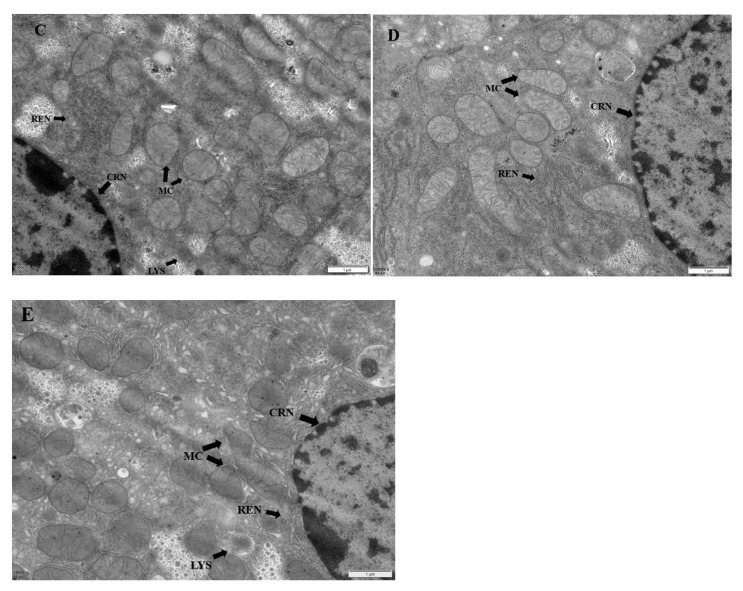
Ultrastructural image of liver; magnification: 1 µm, 15,000×. MC: mitochondria; CRN: nucleus; REN: rough endoplasmic reticulum; LYS: lysosomes. (**A**) Structure of hepatocytes in the CON group. (**B**) Structure of hepatocytes in the F group. (**C**) Structure of liver cells in the LT group. (**D**) Structure of liver cells in the MT group. (**E**) Structure of liver cells in the HT group.

Hepatic ultrastructural changes are shown in Figure 6. In the CON group, the nuclei of the hepatocytes were complete without nuclear deformation or nuclear rupture. The organelles, including the mitochondria, ribosomes, endoplasmic reticulum, and lysosomes, were intact within the cytoplasm. The rough endoplasmic reticulum (RER) was affirmed in layers. There was no evidence of mitochondrial swelling or rupture (Figure 6A). In group F, the hepatocytes showed significant pathological changes, such as deformed or ruptured nuclei, lipid droplets deposited within the cytoplasm, mitochondrial swelling, sharp fractures, dissolution, whitish areas, and increased lysosomes (Figure 6B). However, the pathological changes were mitigated in all TPM treatment groups, especially in the HT group (Figure 6C–E).

### 2.7. mRNA Expression

**Figure 7:**
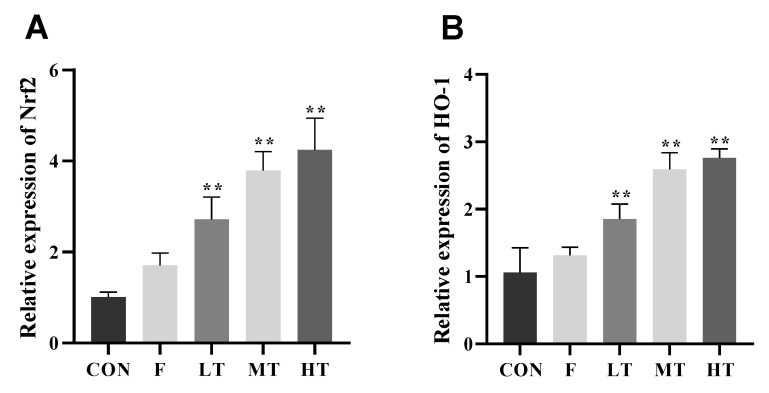

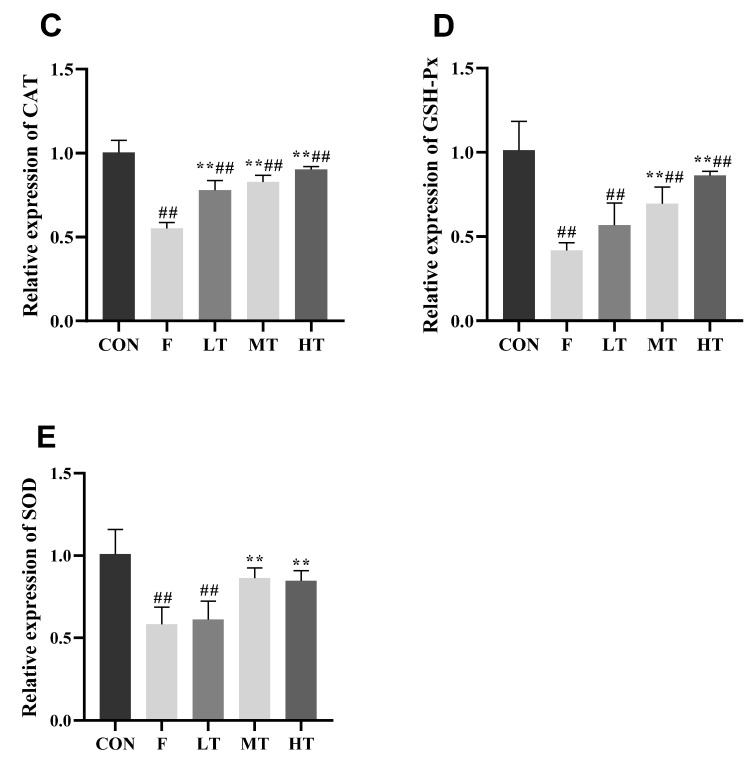
The relative expression of Nrf2, HO-1, CAT, GSH-Px, and SOD mRNA in the liver was measured. (**A**) Nuclear factor E2-related factor 2 (Nrf2), (**B**) heme oxygenase-1 (HO-1), (**C**) the enzyme catalase (CAT), (**D**) glutathione peroxidase (GSH-Px), and (**E**) superoxide dismutase (SOD). ** significantly different compared with group F, *p* < 0.01; ## significantly different compared with group CON, *p* < 0.01.

The effects of the TMP treatment on the Nrf2 expression are shown in Figure 7. The expression levels of CAT, GSH-Px, and SOD decreased significantly in group F compared to group CON (*p* < 0.01). The expression levels of Nrf2, HO-1, CAT, GSH-Px, and SOD in the MT and HT groups were significantly higher than those of group F (*p* < 0.01).

## 3. Materials and Methods

### 3.1. Chemicals

Sodium fluoride (NaF), sodium pentobarbital (C_11_H_17_N_2_NaO_3_), TMP, alanine aminotransferase (ALT, C009-2-1), aspartate aminotransferase (AST, C010-2-1), malondialdehyde (MDA, A003-1-2), reduced glutathione (GSH, A006-2-1), total superoxide dismutase (T-SOD, A001-1-2), and total antioxidant capacity (T-AOC, A015-3-1) were purchased from Jiancheng Bioengineering Institute, Nanjing, China (kit name: PrimeScriptTM RT Reagent KitRR086A). SYBR R Premix Ex TaqTM II (cat. No. RR920A) and RNAiso Plus (cat. No. 9178) were provided by Takara Biotechnology Co., Ltd. (Dalian, LN, China).

### 3.2. Experimental Animals and Methods

The use of animals was approved by the Animal Protection and Use Committee of Sichuan Agricultural University (approval No.: dyy-2021203010). Sixty four-week-old male Institute of Cancer Research (ICR) mice without the carriage of pathogenic bacteria and with a body weight from 18 to 22 g were used in this study (Pegatron Biotech Co., Ltd., Chengdu, SC, China; animal license No.: SCXK (chuang) 2020-030). The mice were housed in a standard SPF facility with a controlled room temperature of 25 ± 2 °C under a 12 h light–dark cycle and fed with a standard pellet diet and water ad libitum. The subjects were fed adaptively for one week and then randomly divided into 5 groups (n = 12 in each group): (1) a control group (CON); (2) a fluorine (alone) group (F), (3) a low-dose TMP group (LT) that received 40 mg/kg of TMP; (4) a medium-dose TMP group (MT) that received 80 mg/kg of TMP; (5) and a high-dose TMP group (HT) that received 160 mg/kg of TMP [26]. Previous studies in our laboratory have demonstrated that the doses of TMP used in this test are safe for mice. TMP was dissolved in distilled water and administered to mice using a gastric tube for 14 days in the three TMP groups. The mice in the control and model groups were administered the same volume of distilled water for 14 days. After the last administration, the mice in the model group and the TMP groups were given NaF dissolved in distilled water (35 mg/kg of body weight) [27] after the last treatment to induce acute hepatopathy; the same volume of distilled water was administered intragastrically to the control group. A total of 24 h after the fluoride challenge, the mice were administered 80 mg/kg of 2% pentobarbital sodium intraperitoneally, cervical dislocation was performed while the mice were anesthetized and already unconscious, and the mice were sacrificed. Blood samples were collected from the orbital venous plexus of the mice. The blood was collected in a sterile 1.5ml EP tube without added anticoagulants, and the serum was subsequently isolated. Serum alanine aminotransferase (ALT), aspartate aminotransferase (AST), and biomarkers of oxidative stress were measured. The livers were harvested, weighed, and stored for further histopathological and biochemical analyses, including an analysis of the histopathological changes, ultrastructural changes, biomarkers of oxidative stress, and gene expressions.

### 3.3. Body Weight, Organ Index, and Liver Index

Body weights were measured and recorded weekly. At the end of the experiment, the brain, heart, liver, kidneys, lungs, and spleen of each subject were collected, weighed, and photographed. The organ index was calculated using the following formula [28]:

Organ index (%) = Organ weight (mg)/body weight (g) × 100%(1)

### 3.4. Detection of Serum Levels of ALT and AST

Blood samples were collected from the mice and stored at 4 °C for 2 h, then centrifuged at 3000 rpm for 10 min. The serum alanine aminotransferase (ALT) and aspartate aminotransferase (AST) levels were measured according to the user’s manual from the manufacturer Nanjing Jiancheng Bioengineering Institute (Nanjing, JS, China).

### 3.5. Detection of Serum Levels of Oxidative Stress Biomarkers

Blood samples were collected from the mice and left to stand at 4 °C for 2 h, followed by centrifugation at 3000 rpm for 10 min to obtain the serum. The total superoxide dismutase (T-SOD) activity, total antioxidant capacity (T-AOC), glutathione (GSH) content, and malondialdehyde (MDA) content were measured using a biochemical reagent kit provided by China Jiancheng Bioengineering Institute (Nanjing, JS, China).

### 3.6. Detection of Hepatic Oxidative Stress Biomarkers

Liver tissues were rinsed in an isotonic saline solution at 4 °C, weighed, and homogenized in an ice-cold isotonic saline solution in a ratio of 1:9 (*w*/*v*), followed by centrifugation (3500 rpm, 10 min at 4 °C). The suspension was collected for a measurement of the total protein concentration using the Bradford protein assay. The hepatic levels of T-SOD, T-AOC, GSH, and MDA were detected using a biochemical reagent kit provided by China Jiancheng Bioengineering Institute (Nanjing, JS, China).

### 3.7. Hepatic Histopathological Changes

The remaining liver tissues were prepared for histopathological examination. The liver tissue was trimmed neatly and rinsed with water for 4 h, an alcohol gradient dehydration was performed, and the dissolved paraffin was infiltrated into the tissue blocks, which became hard wax blocks after cooling. The wax blocks were placed on a slicer (Leica, Heidelberg, Germany), cut into 5 μm slices, and transferred to slides. The wax blocks were placed in a temperature box at 60 °C for 2 h and dried, the paraffin sections were immersed in hematoxylin for 5 min followed by eosin for 2 min, and the slides were dehydrated and sealed with a neutral resin. A Leica DM1000 microscope (Leica, Heidelberg, Germany) was used to observe the histopathological changes [29].

### 3.8. Ultrastructural Changes to Hepatocytes

Samples of the liver preserved in glutaraldehyde were taken and ultrathin sections were made according to standard protocols for an examination of ultrastructural changes using a JEM-1400-FLASH transmission electron microscope (Jeol, Japan).

### 3.9. RNA Extraction and qRT-PCR

The liver samples were stored at −80 °C for RNA extraction. The total liver RNA was extracted using RNAiso Plus (9108, Takara, Otsu, Japan) by following the manufacturer’s instructions. cDNA was synthesized simultaneously using 1 ug of RNA with the Prim-Script TM RT Reagent Kit (9108, Takara, Japan) and the SYBR Premix Ex Taq TM II Kit (9108A, Takara, Japan). The expression levels of Nrf2, HO-1, CAT, GSH-Px, and SOD-1 were detected by real-time quantitative PCR (Bio-Rad, Hercules, CA, USA). β-actin was applied as a housekeeping gene for normalization. The RNA expression levels were calculated using the 2^−ΔΔCT^ method [30]. The primers used in this study were designed by PREMIER 5 (PREMIER Biosoft International, Palo Alto, CA, USA) and are shown in Supplementary Data Table S1. 

### 3.10. Data Statistics and Analysis

All data were analyzed using SPSS 27.0 (SPSS Inc., Chicago, IL, USA). A one-way analysis of variance (ANOVA) and Tukey’s multiple comparison test were used to determine the overall differences among groups. GraphPad Prism 8.0.2 (GraphPad Software, San Diego, CA, USA) was used for graphing. All parameters were expressed as the mean ± standard deviation. Significance was recognized when *p* < 0.05.

## 4. Discussion

Fluorine is a natural element in the environment, and fluoride has been used extensively in daily life, such as in sewage treatments. However, excessive exposure to fluoride leads to fluorosis, resulting in severe hepatopathy. Fluorosis-induced hepatopathy has been reported to be associated with reactive oxygen species (ROS) and the activation of oxidative stress. Excess ROS cause cellular damage due to oxidative stress reactions [31]. TMP is an active ingredient in a traditional Chinese medicine extracted from chuanxiong, which has been reported to be an effective antioxidant and anti-inflammatory agent. TMP has been used to treat liver issues, such as ischemia-reperfusion injury, hepatic fibrosis, septic liver injury, and so on [23,24,32].

The results of the current study reveal that an overdose of fluoride induces liver damage. The serum levels of alanine aminotransferase (ALT) and aspartate aminotransferase (AST) are two important intracellular biomarkers for assessing liver function [29]. The serum levels of ALT and AST increase during hepatocyte injury due to the increased permeability of the cell membranes [33,34]. T-SOD is an antioxidant that effectively scavenges free radicals and alleviates cellular oxidative damage. GSH is another antioxidant synthesized in low-molecular-weight cells that inhibits the production of free radicals. Nonetheless, MDA is an indicator of lipid peroxidation and indirectly reflects an increase in the free radical content during oxidative stress. Furthermore, T-AOC represents the total antioxidant capacity of the body [35,36]. When oxidative stress is in progress, reactive oxygen species (ROS) activate the chain reactions of antioxidants, leading to an elevation in the amount of T-SOD and GSA and a reduction in the level of T-AOC. Meanwhile, the serum content of MDA increases. The results of this study are in line with ROS-triggered antioxidant chain reactions after fluoride-challenged hepatopathy. After the fluoride challenge, the serum levels of ALT, AST, and MDA increased significantly; however, the serum levels of T-SOD, GSH, and T-AOC decreased significantly, which is in accordance with the findings of previous studies [16,35,37]. Among the treatment groups, especially in the MT and HT groups, the levels of T-SOD, GSH, and T-AOC increased, whereas the content of MDA decreased, which suggests that the intervention of TMP alleviated the fluoride-induced oxidative stress and hepatic injury.

Histopathological changes directly reflect cellular damage, which is helpful for evaluating the severity of organ damage. The severity of hepatocyte damage predisposes the cells to progressive ROS-associated damage. The results of this study show that the fluoride treatment induced hepatopathy because of significant observations of hepatocyte vacuolation and swollen mitochondria, indicating severe ongoing oxidative stress after the fluoride challenge. The findings in this study are consistent with those of previous reports [28,38,39]. The interventions of different dosages of TMP alleviated the severity of the hepatocyte damage in the current study, especially in the MT and HT groups. The morphologies of the hepatocytes, including the mitochondria, in these groups were relatively normal compared to those of group F, indicating the potential protection by TMP against fluoride-induced hepatopathy.

Nuclear factor E2-related factor 2 (Nrf2) is ubiquitous in mammalian cells; it combines with antioxidant counter-response factors and triggers the transcription and expression of antioxidant enzymes such as superoxide dismutase (SOD), glutathione peroxidase (GSH-Px), the enzyme catalase (CAT), etc.; however, it is inhibited during oxidative stress. Nrf2 has been reported to be involved in regulating the oxidative balance and protecting antioxidants and phase II detoxification reactions in organisms [40]. The expression of heme oxygenase-1 (HO-1) is regulated by Nrf2, which promotes heme degradation. Physiologically, the expression of Nrf2 is regulated by the Keap1 protein in the cytoplasm; however, Nrf2 is uncoupled from Keap1 and rapidly translocated to the nucleus when oxidative stress occurs, due to the Nrf2-dependent cellular defense mechanism, which activates the transcription of HO-1, SOD, CAT, and GSH-Px in order to enhance cellular antioxidant capacities. The Nrf2/HO-1 signaling pathway is critical for improving the capability of organisms to defend against oxidative-stress-induced cellular damage, and it has been recognized as one of the most important endogenous antioxidant signaling pathway in mammalians [41]. The results of the current study show that the hepatic mRNA expression levels of Nrf2 and HO-1 increased concurrently with decreased expression levels of SOD and CAT in group F, suggesting that acute excessive fluoride intake led to oxidative stress and a compromised ability to scavenge free radicals. Previous studies have shown that fluorosis activates the Keap1–Nrf2 signaling pathway to prevent oxidative-stress-induced hepatic or renal injuries in rats with chronic fluorosis [1,16]. Nevertheless, fluoride-induced oxidative stress promotes the expression of intracellular ROS, inducing peroxiredoxin-dependent unfolded protein responses and triggering the autophosphorylation of Nrf2 [42], which reduces the expression of downstream genes of the Nrf2 pathway and exacerbates oxidative-stress-induced hepatopathy. According to the results of this study, the expression levels of genes associated with the Nrf2 pathway in the liver are enhanced by TMP, suggesting that TMP is effective at promoting the expression of Nrf2-pathway-associated genes by activating the Nrf2/HO-1 signal pathway and facilitating the elimination of excessive ROS in fluoride-induced hepatopathy in mice. This finding is consistent with those of previous studies [23,24,26,43]. Furthermore, it has been reported that TMP significantly enhances Nrf2 expression and nuclear translocation in hepatic stellate cells (HSCs), but in the case of an Nrf2 gene knockdown, the improvement effect of TMP on the serum enzyme activity, the liver histological structure, serum and liver pro-inflammatory cytokine levels, intrahepatic inflammatory cell infiltration, and the anti-fibrosis effect of TMP are also abolished, suggesting that TMP relies on the Nrf2 pathway to play a role in liver oxidative stress and liver fibrosis, and that it improves the expression of Nrf2-pathway-associated genes [44].

## 5. Conclusions

In conclusion, when ICR mice consume a large amount of fluoride over a short period of time, the Nrf2 pathway is activated as a protective mechanism for the liver. Because of the accumulation of ROS in the liver of ICR mice after the fluoride intake, the expression of Nrf2-pathway-related genes is inhibited, and the antioxidant function of the liver is reduced, leading to fluorosis liver injury in ICR mice. TMP ameliorates the fluoride-induced hepatopathy in male ICR mice by promoting the expression of Nrf2 pathway genes, enhancing the hepatic levels of SOD and GSH, and reducing the hepatic level of MDA; overall, the intervention of TMP enhances the hepatic antioxidant capacity. The results of the current study help us to understand the pathophysiological changes of fluoride-induced hepatopathy and the basic mechanisms of action of TMP in alleviating fluoride-induced hepatopathy. However, this trial only explored the protective mechanism of TMP under acute fluorosis; the pathogenesis of chronic fluorosis and the detailed mechanisms of action of TMP in treating fluoride-induced hepatopathy need to be explored further.

## Data Availability

The raw data supporting the conclusions of this article will be made available by the authors, without reservation.

## Supplementary Materials

The following supporting information can be downloaded at: https://www.mdpi.com/article/10.3390/molecules28124849/s1. Table S1: qRT-PCR primer sequence list.

## Sample Availability

Not available from the authors.
