# Supplementary material for: The Pathophysiological Changes and Clinical Effects of Tetramethylpyrazine in ICR Mice with Fluoride-Induced Hepatopathy
Source: Molecules. 2023 Jun 19;28(12):4849. doi: 10.3390/molecules28124849 (PMC10302661; doi:10.3390/molecules28124849)
Supplement: Supplementary file 1 [file molecules-28-04849-s001.zip › molecules-2407361-supplementary.pdf]

Table S1. qRT-PCR Primer Sequence Listing.

Gene	Accession number	Primer sequence (5′-3′)	Product length(bp)
Nrf2	NM-010902.4	F: AGCGGTAGTATCAGCCAR: GCCCAGTCCCTCAATAGC	150
HO-1	NM-010442.2	F: TGTTGCGCTCTATCTCCR: GTACACATCCAAGCCGAG	136
CAT	NM-009804.2	F: TTCCTGAGCAAGCCTTCR: CACATGAATGGCTATGGATCACA	138
GSH-Px	NM-008160.6	F: TCTCTTCATTCTTGCCATR: GACTACACCGAGATGAACGA	112
SOD1	NM-011434.2	F: CACCTTTGCCCAAGTCAR: TCCATTGAAGATCGTGT	97
β-Actin	NM-007393	F: CGCTCGTTGCCAATAGTGR: GCTGTGCTATGTTGCTCTAG	117
